# A Novel MYO5A Mutation (c.3508C>T) in a 27‐Month‐Old Girl With Hypotonia and Developmental Delay: Expanding the Phenotypic Spectrum of Griscelli Syndrome Type 1

**DOI:** 10.1155/crii/3883928

**Published:** 2026-06-28

**Authors:** Davoud AmirKashani, Soudabeh Hosseini, Marziyeh Mojbafan, Niusha Sharifinejad, Sima Bahrami

**Affiliations:** ^1^ Aliasghar Clinical Research Development Center, Department of Pediatrics, School of Medicine, Iran University of Medical Sciences, Tehran, Iran, iums.ac.ir; ^2^ Aliasghar Children Hospital, School of Medicine, Iran University of Medical Sciences, Tehran, Iran, iums.ac.ir; ^3^ Department of Medical Genetics, School of Medicine, Iran University of Medical Sciences, Tehran, Iran, iums.ac.ir; ^4^ Non-communicable Diseases Research Center, Alborz University of Medical Sciences, Karaj, Iran, abzums.ac.ir; ^5^ Department of Allergy and Clinical Immunology, Aliasghar Children Hospital, School of Medicine, Iran University of Medical Sciences, Tehran, Iran, iums.ac.ir

**Keywords:** Elejalde syndrome, Griscelli syndrome, Griscelli syndrome type 1, GS, MYO5A

## Abstract

**Background:**

Griscelli syndrome (GS) is a rare autosomal recessive disorder, characterized by pigmentary dilution of hair and skin. There are three known types of this disease. GS type 1 (GS1), due to mutations in *MYO5A*, is associated with neurological impairment and hypotonia, while GS type 2 (GS2; *RAB27A* variants) involves immune dysregulation (hemophagocytic lymphohistiocytosis [HLH]) and GS type 3 (*MLPH* variants) causes isolated pigmentary defects.

**Case Presentation:**

We report a 27‑month‑old Afghan girl, born to consanguineous parents, presenting with developmental delay and hypotonia without the classic silvery hair. A complete evaluation was performed to determine the cause of the developmental delay. Whole exome sequencing revealed a novel homozygous MYO5A variant (c.3508C >T), confirming GS1. Despite the absence of the characteristic silvery hair, microscopic hair examination of the patient showed melanin distribution abnormalities.

**Conclusion:**

This case highlights the phenotypical variability in GS1 and emphasizes on the importance of considering genetic testing even in atypical presentations. Exploring these novel mutations can expand the known genotype/phenotype spectrum of GS1.

## 1. Introduction

Griscelli syndrome (GS) is a rare autosomal recessive disorder characterized by pigmentary dilution of the skin and hair. Three distinct classifications of GS have been described according to the underlying genetic defect and clinical features, all sharing pigmentary abnormalities from birth or very early infancy [1]; type 1, resulting from mutations in the *MYO5A* gene, is mainly associated with hypotonia and severe neurodevelopmental delay [2]. Type 2, caused by mutations in *RAB27A*, typically presents with immunological dysfunction and is often recognized during episodes of hemophagocytic lymphohistiocytosis (HLH) [3]. Type 3, related to variants in the *MLPH* gene, manifests only with pigmentary changes without neurological or immunological involvement [4]. So far, the majority of reported cases are categorized as type 2, while type 1 and 3 remain exceedingly rare. While GS type 2 (GS2) is often fatal and the only cure is hematopoietic stem cell transplantation (HSCT), the treatment for GS type 1 (GS1) is only symptomatic. There is no specific management for GS3 [1]. Table 1 provides a better understanding of the different types of GS.

**Table 1:** Clinical and genetic features of Griscelli syndrome subtypes.

Subtype	Gene	Main features	Onset/time course	Prognosis
GS1	*MYO5A*	Neurological impairment, hypotonia, pigmentary abnormalities	Neurological symptoms and pigment changes typically appear at birth or in early infancy	Poor; progressive neurological decline
GS2	*RAB27A*	Immune dysfunction, HLH, pigmentary changes	‐ Pigment changes at birth or early infancy ‐ Immune dysfunction appears in infancy (usually before 2 years) ‐ HLH episodes often occur within the first months to first year of life	Life‐threatening without HSCT
GS3	*MLPH*	Isolated pigmentary defects only	Pigment changes apparent from birth or early infancy	Benign

Abbreviations: GS, Griscelli syndrome; HLH, hemophagocytic lymphohistiocytosis; HSCT, hematopoietic stem cell transplantation.

Here, we present an interesting case of GS1 with neurodevelopmental disorder carrying a novel *MYO5A* (c.3508C >T, p.Gln1170 ^∗^) mutation.

## 2. Case Presentation

**Figure 1:**
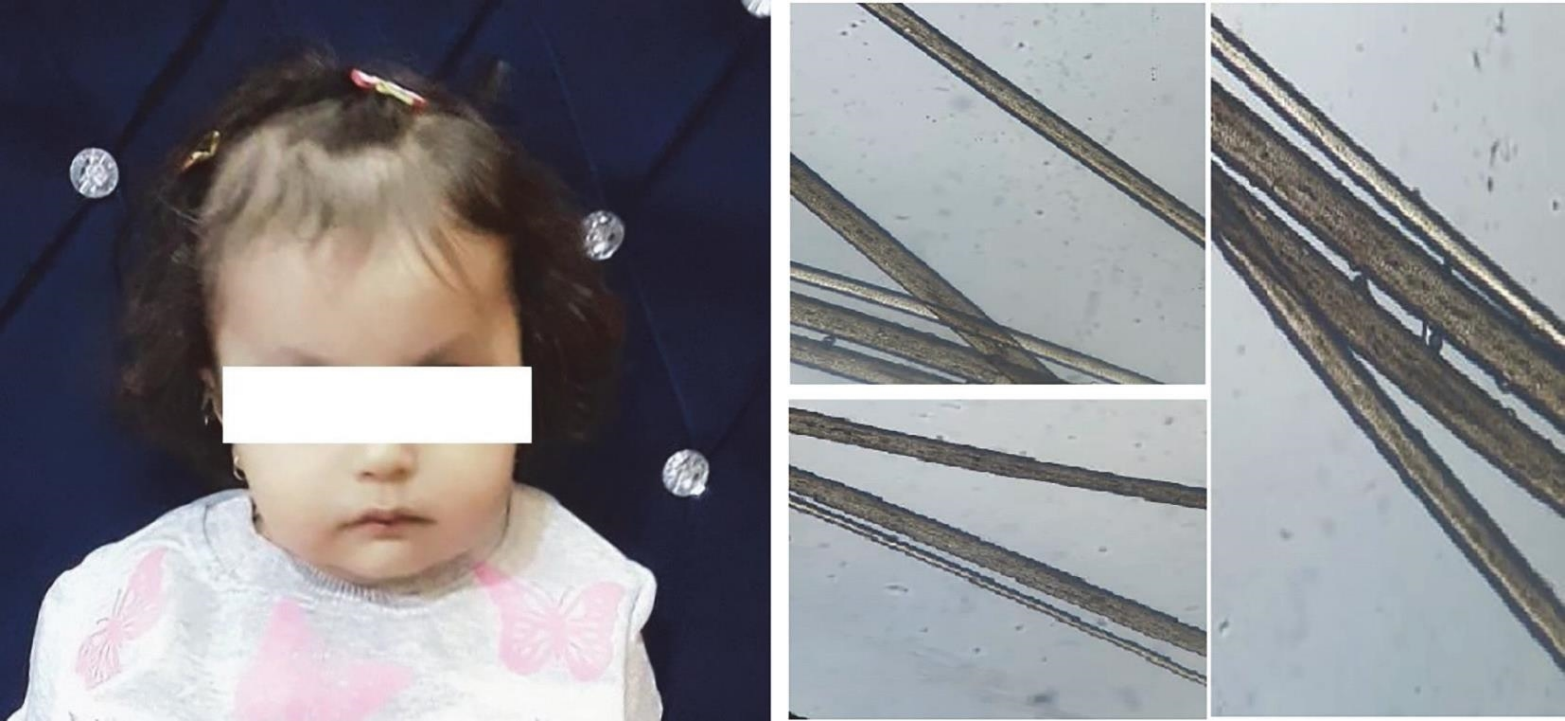
A picture of our patient with Griscelli type 1 syndrome without the classic silvery hair, taken at 27 months of age. On the right side, the picture illustrates the abnormal melanin distribution of our patient’s hair under light microscope. This abnormal pattern has heterogenous pigmentation in both a part of a single strand or adjacent hair strands, deposition of nonuniform melanin along the cortex, and presence of unevenly scattered clumps of melanin granules. The granular pattern is heterogeneous along the cortex, with areas of fine (<2 µm) and coarse (>4 µm) aggregation.

A 27‑month‐old Afghan girl was referred to the immunology clinic. She was the first child of consanguineous parents, born via cesarean section with normal Apgar scores. No previous family history of similar condition, miscarriage, or neonatal death was present. At the time of her first appointment, she had light brown hair, lighter than her parents, which remained the same from birth. Abnormal hypotonia and developmental delay were evident during the examination. Figure 1 illustrates a picture of our patient at 27 months old. Her parents first noticed hypotonia at 2 months of age, and she failed to reach the developmental milestones within the expected timeline. She was unable to maintain head control without support until the age of 7 months. She gradually gained the ability to sit with support after 12 months. By the age of 24 months, she could roll over, stand with support, and uttered a few incomprehensible sounds.

She had no visual impairment or any episodes of seizures up to the age of 27 months. Growth parameters were within the normal limits. At 1 year of age, she was evaluated for anemia, and a diagnosis of minor thalassemia was considered. At 24 months, a comprehensive evaluation for neurometabolic disorders was performed to determine the probable cause of the developmental delay. The immunological investigations showed no immunological or cytotoxic defects. Whole‐exome sequencing revealed a novel *MYO5A* mutation (c.3508C >T). This mutation occurs on exon 26 out of 42 of this gene and result in a premature stop codon that may produce a truncated protein. This variant is a likely pathogenic mutation according to The American College of Medical Genetics and Genomics (ACMG) guideline; it is a null variant and a loss of function mutation, which is a known mechanism of disease in this gene (PVS1). Additionally, this variant has an extremely low frequency in different databases like 1000 Genome and gnomAD (PM2). Considering the probable diagnosis of GS, her hair was further examined, which revealed an abnormal melanin distribution (Figure 1). Both parents were found to be heterozygous carriers of the *MYO5A* (c.3508C >T) mutation.

GS1, as an autosomal recessive disorder, carries a 25% recurrence risk in future pregnancies for consanguineous couples. Genetic counseling was provided to the family, including discussing prenatal and preimplantation genetic diagnosis options.

## 3. Conclusion

In this report, we present a 27‐month‐old child with hypotonia and neurodevelopmental delay as the main manifestations. Unlike the typical cases of GS, our patient did not initially display albinism or silver hair, which delayed the diagnosis of a melanocytic disorder. Only after genetic testing, a novel *MYO5A* mutation (c.3508C >T, p.Gln1170 ^∗^) was revealed. Later, the microscopic examination of her hair confirmed an abnormal melanin distribution, and the diagnosis of GS1 was established. This is the first reported case of GS1 presenting without the characteristic silver hair or pale bronze skin, only with a mild abnormality in melanocyte distribution.

The *MYO5A* gene encodes a protein of the myosin family, which plays a crucial role in intracellular transport. Myosin proteins are also involved in maintaining cellular structure and facilitating overall cell movement through interactions with actin filaments [5], thus explaining the severe hypotonia in GS1 patients. In melanocytes, these functions are particularly important, as pigmentation relies on melanosomes, the intracellular organelles that contain melanin [6]. MYO5A interacts with proteins encoded by the *RAB27A* and *MLPH* genes to transport melanosomes within melanocytes. Disruption of this protein complex leads to pigmentation abnormalities, which are observed in all types of GS [7]. However, the *MYO5A* mutation (c.3508C >T, p.Gln1170 ^∗^) in our patient represents a previously unreported variant and may explain the milder pigmentation phenotype in our patient.

MYO5A also plays a critical role in the nervous system. The intracellular movement of organelles within neurons, as well as their exocytosis, depends on functional myosin 5A. Therefore, mutations in *MYO5A* can result in severe neurological dysfunction [8], which is in line with the developmental delay in our case. In 1979, Elejalde described a syndrome characterized by skin and hair pigmentation abnormalities accompanied by profound neurological impairment and normal immune function [9]. Subsequent studies identified mutations at the 15q21 locus, which were later linked to *MYO5A*, leading to the reclassification of these patients as GS1 [10]. Interestingly, Cetica et al. [11] also reported GS2 patients with familial HLH and biallelic mutations in RAB27A in the absence of albinism. Their gene sequencing later revealed mutations that only disrupted the role of Rab27a in cytotoxic immunity [11].

This study illustrates the clinical variability of GS1 and underscores the importance of genetic testing in children with unexplained developmental delay and hypotonia. Our patient showed that GS1 may present with microscopic pigmentary abnormality. In such cases, genetic testing is essential to confirm the diagnosis. Identification of new mutations, such as our novel *MYO5A* mutation, expands the mutational spectrum of GS1 and also genotype–phenotype correlations, resulting in an earlier diagnosis and better management.

**Nomenclature**
GS: Griscelli syndrome
GS1: GS type 1
HLH: Hemophagocytic lymphohistiocytosis
HSCT: Hematopoietic stem cell transplantation
ACMG: The American College of Medical Genetics and Genomics.

## Author Contributions

Davoud AmirKashani performed analysis and interpreted the patient data, Soudabeh Hosseini assisted in patient diagnosis, Marziyeh Mojbafan performed genetical assessment of the patient, Niusha Sharifinejad contributed in manuscript writing and data gathering, and Sima Bahrami was a major contributor in interpretation of patient data and writing the manuscript.

## Funding

No funding was received for this manuscript.

## Disclosure

All authors have read and approved the final manuscript.

## Ethics Statement

The authors have nothing to report.

## Consent

Informed consent was obtained from the parents of the patient prior to being included in the study.

## Conflicts of Interest

The authors declare no conflicts of interest.

## Data Availability

Data sharing is not applicable to this article as no datasets were generated or analyzed during the current study.
